# Deterministic skill of ENSO predictions from the North American Multimodel Ensemble

**DOI:** 10.1007/s00382-017-3603-3

**Published:** 2017-03-13

**Authors:** Anthony G. Barnston, Michael K. Tippett, Meghana Ranganathan, Michelle L. L’Heureux

**Affiliations:** 1 International Research Institute for Climate and Society, The Earth Institute of Columbia University, 65 Route 9W, Palisades, New York, NY 10964 USA; 2 Department of Applied Physics and Applied Mathematics, Columbia University, New York, NY USA; 3 Department of Meteorology, Center of Excellence for Climate Change Research, King Abdulaziz University, Jeddah, Saudi Arabia; 4 Swarthmore College, Swarthmore, PA USA; 5 Climate Prediction Center, National Weather Service, National Oceanic and Atmospheric Administration, National Centers for Environmental Prediction, College Park, MD USA

## Abstract

Hindcasts and real-time predictions of the east-central tropical Pacific sea surface temperature (SST) from the North American Multimodel Ensemble (NMME) system are verified for 1982–2015. Skill is examined using two deterministic verification measures: mean squared error skill score (MSESS) and anomaly correlation. Verification of eight individual models shows somewhat differing skills among them, with some models consistently producing more successful predictions than others. The skill levels of MME predictions are approximately the same as the two best performing individual models, and sometimes exceed both of them. A decomposition of the MSESS indicates the presence of calibration errors in some of the models. In particular, the amplitudes of some model predictions are too high when predictability is limited by the northern spring ENSO predictability barrier and/or when the interannual variability of the SST is near its seasonal minimum. The skill of the NMME system is compared to that of the MME from the IRI/CPC ENSO prediction plume, both for a comparable hindcast period and also for a set of real-time predictions spanning 2002–2011. Comparisons are made both between the MME predictions of each model group, and between the average of the skills of the respective individual models in each group. Acknowledging a hindcast versus real-time inconcsistency in the 2002–2012 skill comparison, the skill of the NMME is slightly higher than that of the prediction plume models in all cases. This result reflects well on the NMME system, with its large total ensemble size and opportunity for possible complementary contributions to skill.

## Introduction

Because the El Niño/Southern Oscillation (ENSO) phenomenon is the strongest driver of climate variability on the seasonal to interannual timescale aside from the seasonal cycle itself (e.g., McPhaden et al. 2006), predictions of the state of ENSO are important for forecasts of seasonal climate anomalies for known seasons and regions of the globe (Ropelewski and Halpert 1987). ENSO is a coupled ocean/atmosphere phenomenon characterized by anomalies in tropical Pacific subsurface and SST, atmospheric circulation and patterns of cloudiness and rainfall (Bjerknes 1969). The quality of predictions of the ENSO state has improved over the decades beginning with the first ones in the 1980s based on simplified coupled ocean–atmosphere physics (e.g., Cane et al. 1986) and advancing into the twenty-first century with comprehensive coupled general circulation models and sophisticated data assimilation techniques to set the initial conditions. Evaluations of the skill of ENSO predictions have been presented periodically along this long developmental path (e.g., Barnston et al. 1994, 1999, 2012; Tippett and Barnston 2008; Tippett et al. 2012; L’Heureux et al. 2016; Tippett et al. 2017).

Here we examine the deterministic predictive skill of a set of some of today’s leading coupled dynamical model predictions of SST in the east-central tropical Pacific Ocean—the Niño3.4 region—known to be representative of the oceanic component of the ENSO state. The models are those of the North American Multi-model ensemble (NMME; Kirtman et al. 2014), and include models from both operational forecast producing centers and research institutions in the U.S. and Canada. This model set contains some, but not all, of today’s leading state-of-the-science models. For example, it excludes the EUROSIP models (e.g., Palmer et al. 2004), which likely have at least comparable if not better predictive skill, including the United Kingdom Meteorological Office (UKMO) model, the European Center for Medium Range Weather Prediction (ECMWF) model,^1^ and the Metéo France model. The Predictive Ocean Atmosphere Model for Australia (POAMA) model, as well as other state-of-the-art models in Japan, China, and some other nations, are also likely competitive.

An outstanding feature of the NMME project is the availability of a homogeneous history of hindcasts on the monthly to interannual time scale spanning the 1982–2010 period. Real-time seasonal predictions in the same format begin in 2011 and continue to the present, as of 2017 (Kirtman et al. 2014). In phase I of the NMME project, predictions of SST, 2-m temperature, precipitation and several additional fields were issued as 1-month averages, and in phase II daily prediction data also began being produced (but not in real-time). Real-time predictions from the NMME models are produced by the 8th day of each month for use in operational monthly and seasonal climate prediction. To ensure homogeneity between the hindcasts and the real-time NMME predictions, efforts were made to produce both sets of outputs as identically as possible. In the case of the CFSv2 model, the real-time ensemble here is constructed using the same pattern of start dates used in the hindcasts, which differs from the real-time sampling of start dates used operationally by CPC (Emily Becker, personal communication). Additional details on the ensemble numbers and start times are provided in Tippett et al. (2017). We always use 24 ensemble members (never more, even though 28 are available for November starts), and use as many members as possible for the tenth lead, for which fewer than 24 members are available during the real-time period.

The purpose of this study is to assess the quality of the NMME predictions of the ENSO-related SST anomaly and compare findings with other recent ENSO prediction skill studies. While the deterministic predictive skill of the NMME is addressed here, an assessment of the probabilistic skill of the NMME system is provided in Tippett et al. (2017). NMME model skill is assessed only with respect to the observations, in contrast to studies focusing largely on potential predictability that estimate the upper limit of predictive skill (e.g., Becker et al. 2014; Kumar et al. 2017). Although this study overlaps to some extent with Barnston et al. (2015), the latter focused more on model systematic errors and their correction, while here the deterministic skills of each individual model and the NMME are described more explicitly at all lead times, and model performances compared with those of other sets of model ENSO forecasts both in the recent past and over a longer hindcast period.

## Data and methods

The period of NMME model predictions studied here includes both hindcasts (1982–2010) and real-time predictions (2011–2015), covering a total of 34 years. Included in the analyses are the medium and long lead forecasts extending into 2016, encompassing the strong 2015–2016 El Niño. The hindcasts and real-time predictions of all participating models are conveniently formatted on the same 1 degree grid. Some of the original models of the NMME project have been discontinued or replaced by improved versions of the same basic model. As of late 2016, the participating models, used in this study, are shown in Table 1 along with a few of their basic characteristics, including the shortened model names used in the discussions here. The maximum lead times of the models range from 9 months (for the NASA model) to 12 months for all of the others except for CFSv2, which predicts out to 10 months. The number of ensemble members ranges from 10 for most of the models, to 24 for the CFSv2 model.

**Table 1:** Basic information for the 8 models of the NMME used in the study

Model	Expanded model name	Name used here	No. ensemble members	Max lead (months)
1. CMC1-CanCM3	Canadian coupled model #1	CMC1	10	12
2. CMC2-CanCM4	Canadian coupled model #2	CMC2	10	12
3. COLA-RSMAS-CCSM4	COLA/Univ. Miami/NCAR coupled model	CCSM4	10	12
4. GFDL-CM2pl-aer04	Modified version of GFDL coupled model	GFDL	10	12
5. GFDL-CM2p5-FLOR-A06	Expanded version of GFDL coupled model, FLOR-A06	GFDL-FLOR-A	10	12
6. GFDL-CM2p5-FLOR-B01	Expanded version of GFDL coupled model, FLOR-B01	GFDL-FLOR-B	10	12
7. NASA-GMAO-062012	Modified version of NASA coupled model	NASA	12	9
8. NOAA/NCEP-CFSv2	NOAA/NCEP coupled model	CFSv2	24	10

The gridded NMME hindcast and real-time forecast data used here are available on the International Research Institute for Climate and Society (IRI) Data Library, at http://iridl.ldeo.columbia.edu/SOURCES/.Models/.NMME. The region in the east-central tropical Pacific whose area-average SST predictions are analyzed here is the Niño3.4 region (5°N–5°S, 120°–170°W), which has been shown to be closely related to the overall ENSO state (Barnston et al. 1997). The Niño3.4 index has been used at some operational centers as a key oceanic component of the ENSO state (e.g., Kousky and Higgins 2007), although other centers use other SST indices more heavily (e.g., Japan Meteorological Agency uses Niño3) or a set of several indices together.

The SST observations used here are the Optimum Interpolation SST data version 2 (Reynolds et al. 2002), available at http://iridl.ldeo.columbia.edu/expert/SOURCES/.NOAA/.NCEP/.EMC/.CMB/.GLOBAL/.Reyn_SmithOIv2/.monthly/.sst. The original 360 by 180 one-degree latitude/longitude grid is converted to a 360 by 181 grid to correspond with the NMME data.

The predictive verification measures examined here address the quality of the deterministic SST predictions, those predictions defined by the ensemble mean (representing the forecast signal, and ignoring the uncertainty) of the prediction anomalies for a given model. These measures include (1) mean squared error skill score (MSESS), which is based on a comparison between the mean squared error of the forecasts and that of predictions of the climatological average, and (2) the temporal anomaly correlation between predictions and observations. The dependence of skill on the target season, lead time and individual model are highlighted. Of interest is the skill benefit of the multi-model ensemble (MME) mean prediction as compared with the individual model predictions. Here, the MME is defined as an average of the pooled ensemble member predictions of the individual models. Under this definition, models with larger numbers of ensemble members are weighted more heavily than those with fewer members. Although this is not how the MME is defined at the Climate Prediction Center (where each model’s ensemble mean is given equal weight), we chose this method because assigning as much weight to a model with relatively few members as to a model with a large number of members is expected to diminish the skill of the MME if the model forecasts have similar average skill, as it diminishes the effective number of independent realizations.

Monthly anomalies for each model and for the observations are defined with respect to their own 29-year climatology period of 1982–2010. By using the anomalies in the analyses, mean forecast biases for each forecast start month and lead time are approximately removed for each model.^2^ For skill comparisons with the predictions from models of the IRI/CPC ENSO prediction plume, which are for 3-month averaged SST data, 3-month average anomalies are used for the NMME SST predictions as well.

**Fig. 1:**
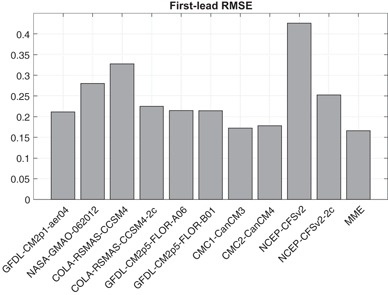
Root-mean squared error (RMSE) of the first-lead (0.5 month) forecasts, 1982–2015

**Fig. 2:**
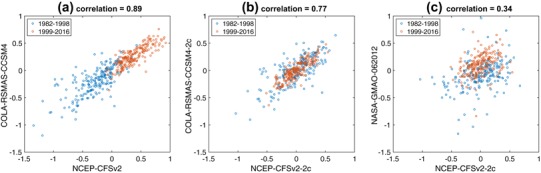
Scatter plots of the first-lead errors (ensemble mean forecast anomaly minus observation anomaly) of **a** CFSv2 versus CCSM4, **b** CFSv2-c2 versus CCSM4-2c, and **c** CFSv2-c2 versus NASA, 1982–2015. *Blue circles* shows forecasts for 1982–1998, and *red circles* for 1999–2016

While eliminating mean bias would normally be expected to result in near-zero mean errors spanning the decades from the early to the later portions of the study period, a different situation is found in the case of two of the NMME models. The root-mean squared error (RMSE) of the ensemble mean forecast anomalies with respect to the observed anomalies for the shortest forecast lead (i.e., the first month) are shown in Fig. 1, averaged over all forecasts during 1982–2016, for each model and for the multi-model ensemble average prediction. Figure 1 indicates that the RMSE of the CFSv2 forecasts is about twice that of most other models, which have RMSE less than 0.25 °C. Pertinent to this high RMSE is the fact that several studies have noted a discontinuity in the forecast bias of the CFSv2 SST hindcasts for the central and eastern tropical Pacific occurring around 1999, which has been related to a discontinuity in the data assimilation and initialization procedure (Xue et al. 2011; Kumar et al. 2012; Barnston and Tippett 2013; Tippett et al. 2017). The time series of the difference between the ensemble mean prediction and the observed anomalies (not shown) indicates that CFSv2 first-lead forecasts tend to be too cool prior to 1999 and too warm after 1999. CCSM4 also has a relatively high first-lead RMSE (Fig. 1), and its first-lead errors are highly correlated with those of CFSv2, though with lower amplitude (Fig. 2, left panel). This behavior is explained by the fact that CCSM4 shares initial conditions with CFSv2 (Kirtman et al. 2014; Infanti and Kirtman 2016), which come from the Climate Forecast System Reanalysis (CFSR; Saha et al. 2010).

One option for treating the discontinuous forecast biases for these two models is to form forecast anomalies using two climatological periods: one for 1982–1998 and one for 1999–2015. The resulting two-climatology forecasts, denoted CFSv2-2c and CCSM4-2c, have lower first-lead RMSE (Fig. 1) and no longer show systematic shifts in their forecast biases near 1998/1999, though the correlation of the first-lead errors remains high because both models are initialized using CFSR (Fig. 2, middle panel). First-lead forecast errors of other models present much lower correlation (e.g., 0.37 for NASA; Fig. 2, right panel) with those of CFSv2-2c. We use these two-climatology versions of CFSv2 and CCSM4 anomaly forecast data hereafter in the analyses of the predictions of individual models and the MME, unless noted otherwise.

In this study, cross-validation is not used in assessing the model skills. Reasons for not using cross-validation are (1) the skill in prediction of the ENSO state is often at least moderately high, and the difference in skill with versus without cross-validation is small at higher skill levels; and (2) we look to assess the *relative* skills of one model versus another or one MME against another.

## Results

The results are presented in three parts: (1) illustrative examples of direct comparisons between NMME model predictions and the corresponding observations; (2) summary scores from objective deterministic verification of the predictions against observations, and (3) comparison of verification results for NMME with alternative dynamical model sets from other recent studies.

### NMME predictions and their corresponding observations

**Fig. 3:**
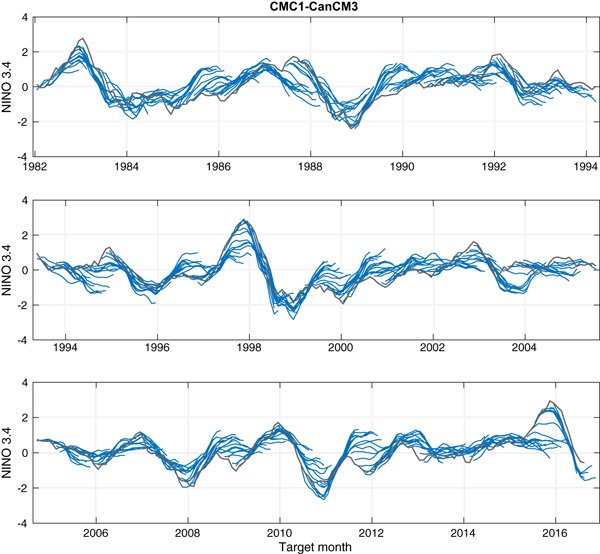
Forecasts (*blue*) of the CMC1 model and observations (*gray*) throughout the study period. A forecast trajectory is shown for start times spanning all months

**Fig. 4:**
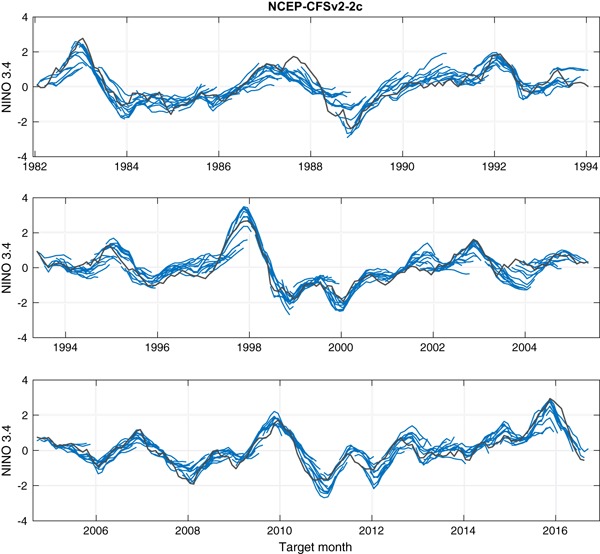
Forecasts (*blue*) of the CFSv2 model and observations (*gray*) throughout the study period. A forecast trajectory is shown for start times spanning all months. The forecast anomalies are with respect to climatologies spanning two base periods having differing forecast biases (see the text)

**Fig. 5:**
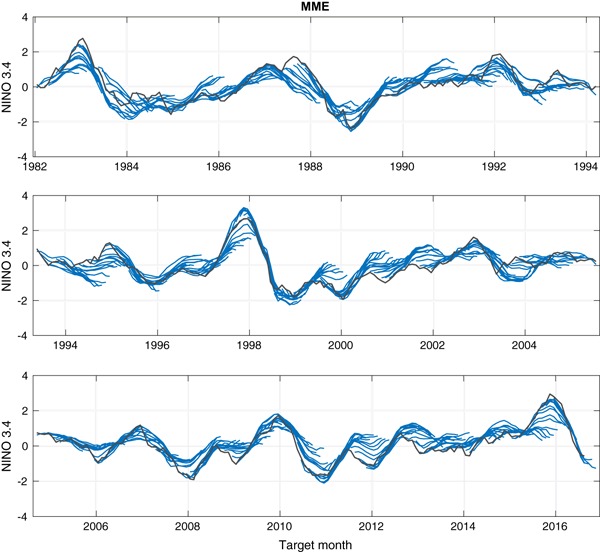
Forecasts (*blue*) of the MME and observations (*gray*) throughout the study period. A forecast trajectory is shown for start times spanning all months

Figures 3 and 4 show time series comparing the predictions of two of the eight NMME models (CMC1 and CFSv2, respectively) with the observations, and Fig. 5 shows likewise for the MME predictions. These three figures illustrate the basic data analyzed in this study. The predictions are shown as lines from each consecutive start month extending to the maximum lead. At the longest leads, the MME forecast is defined using forecasts from only the models whose forecasts extend to those leads.

Figures 3 and 4 show predictions that are generally in the anomaly direction matching that of the observations, but this correspondence weakens with increasing lead. Some ENSO events were relatively well predicted by both models and by the MME even at long leads, as for example the El Niño of 2009–2010. Where model errors are seen, some of them are similar between the two models while others occur in just one of them. For example, some underestimation of the strength of the 2015–2016 El Niño event is seen in both individual models and also in the MME, particularly at longer leads. On the other hand, the weak El Niño of 1994–1995 was not predicted well at long leads by the CMC1 (Fig. 3) nor by the MME (Fig. 5), but was handled better by the CFSv2 model (Fig. 4).

**Fig. 6:**
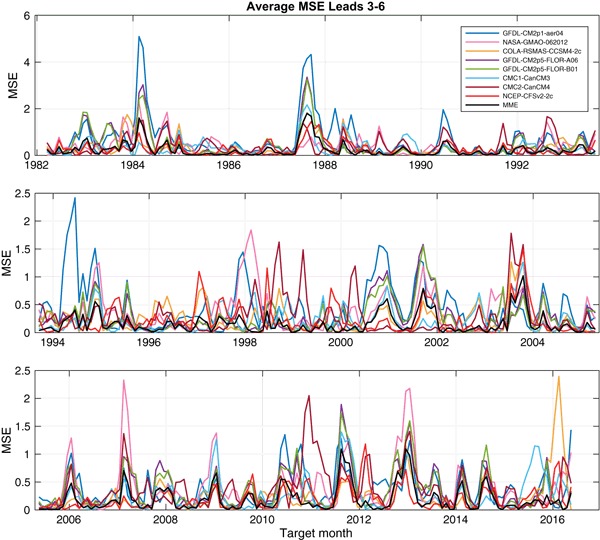
Time series of mean squared errors of each individual NMME model and of the MME, averaged over the third to sixth lead (2.5 to 5.5 month leads). Note that the top row has larger scale, to accommodate occasional very large squared errors in the 1980s

To extend the view of model errors to all of the models, Fig. 6 shows times series of the mean squared error (MSE) of each of the 8 individual models and of the MME, averaged over moderate lead times spanning from the third to the sixth month, over the 1982–2016 study period. Figure 6 reveals that all of the individual models have times when their errors are the greatest among the model set, and when they are the smallest. Times of particularly poor model performance are identifiable, such as the large error of the GFDL model in early 1984 and late 1987. In both cases, the error was in the direction of too cold a forecast (not shown) following an El Niño event in which during the year following the El Niño, the SST did not tend to be cooler than average as usually occurs. A cold error in this same circumstance occurred to the greatest extent in the CFSv2 (Fig. 4) and the CMC2 (not shown) models—which we will see are the two generally highest performing models of the eight—in late 2003 following the 2002–2003 El Niño. The MSE performance of the MME, while generally at an intermediate level during many of the target months, is often smaller than the mean MSE of the individual models. This can be explained by the fact that, in many but not all cases, the errors of some of the individual models are of opposing signs, creating a smaller magnitude of error when averaged in the MME prediction and making the MME an effective final forecast product. Additionally, the different models can contribute additional forecast signals (DelSole et al. 2014). The advantage offered by the MME is also shown in Fig. 1 for the shortest lead time, as the MME has a slightly smaller RMSE result than the most skillful of the individual models (in this case, CMC1 and CMC2). Such a MME benefit was found in other multi-model studies (e.g., Peng et al. 2002; Kharin and Zwiers 2002; Palmer et al. 2004; Kirtman et al. 2014).

**Fig. 7:**
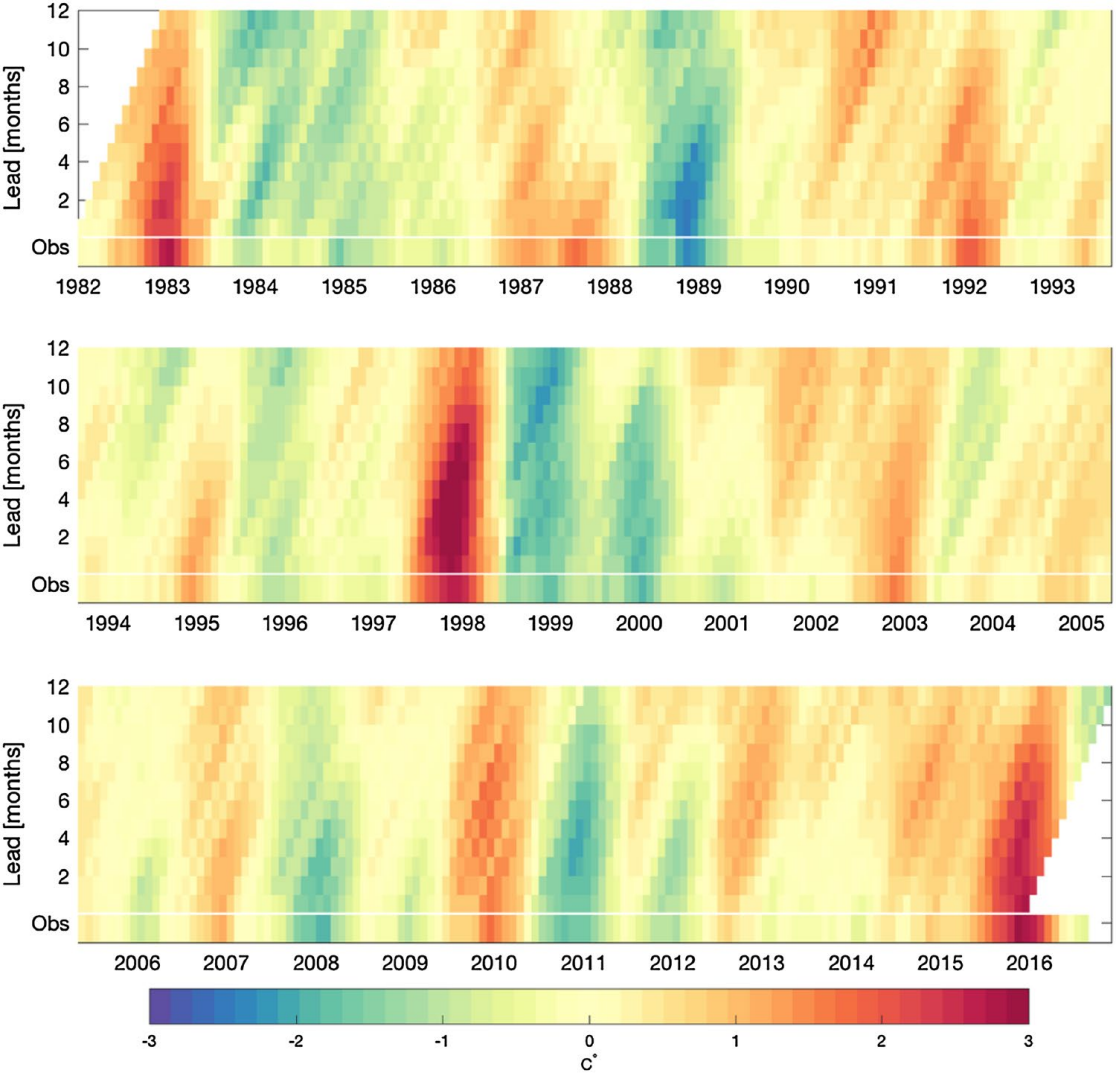
MME forecasts, by target month, as a function of lead time (hence, forecasts were made increasingly earlier than the target month toward the *upper* part of panel). The observations are shown at the *bottom* of the panel

**Fig. 8:**
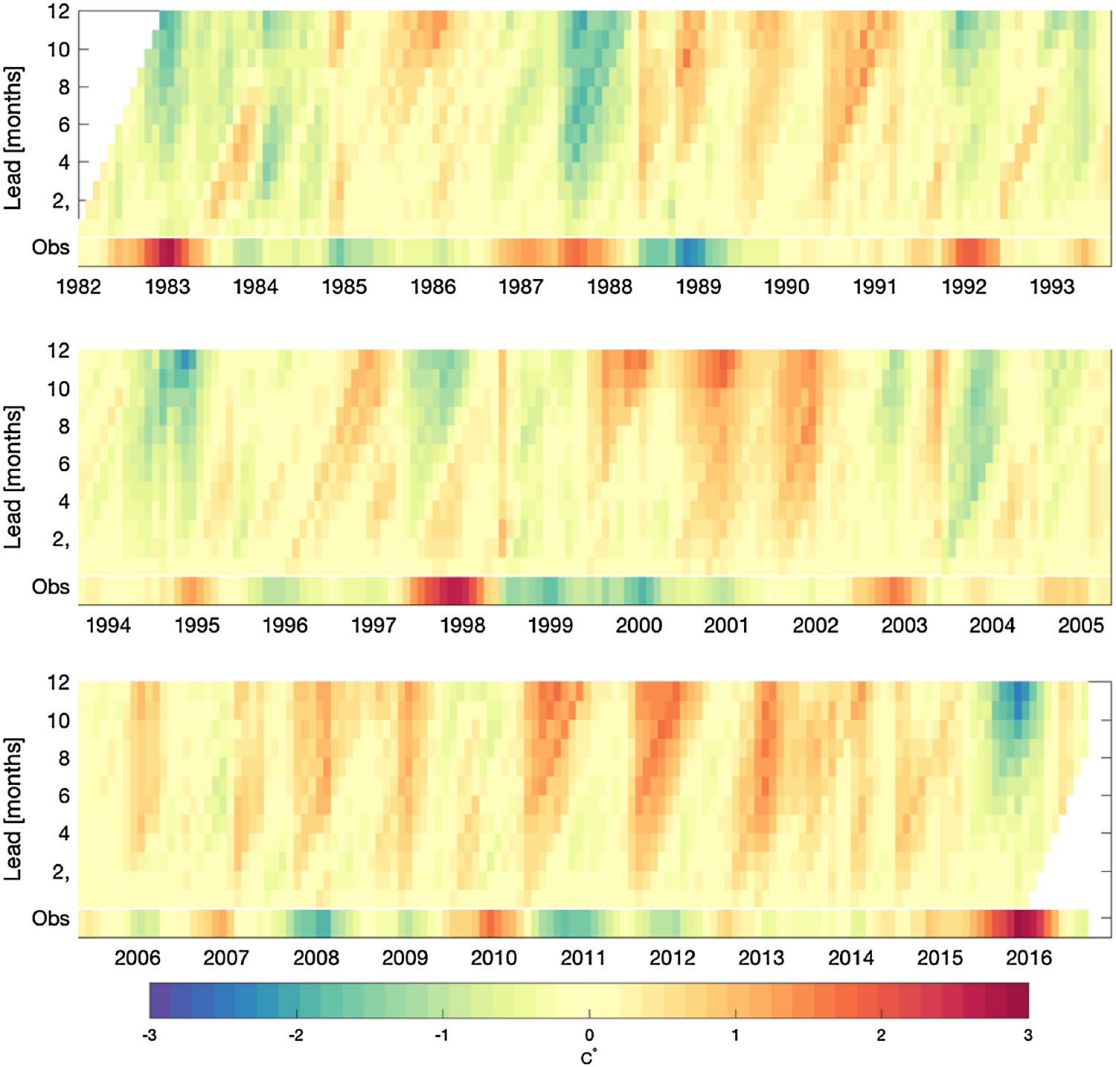
MME error, by target month, as a function of lead time (hence, errors are for forecasts made increasingly earlier than the target month toward the *upper* part of panel). The observations are shown at the *bottom* of the panel

Figure 7 shows a history of the MME forecast, by target month and lead time, throughout the 1982–2016 period, and Fig. 8 shows the MME forecast error in the same format. In both figures, the corresponding observations are shown along the bottom of the panels. Lead time is defined as the number of months between the forecast start time (at the beginning of a month) and the center of the month being predicted. For example, for a forecast starting at the beginning of January, the forecast for January has a 0.5-month lead, for February a 1.5-month lead, etc., spanning to 11.5-month lead for most of the models and for the NMME. Figure 7 shows that the forecasts generally capture the major fluctuations of the Niño-3.4 SST successfully, and to greater extents with decreasing lead time. The strong El Niño events of 1982–1983, 1997–1998 and 2015–2016 were well predicted, but were underestimated to greater extents as lead time increased. The same holds true for the strongest La Niña events of 1988–1989, 1998–1999, 1999–2000 and 2010–2011. Some false alarms are noted at longer lead times, such as the El Niño events predicted for 1990–1991 and 2012–2013 that did not occur. The MME error profile (Fig. 8) highlights such false alarms as well as the underpredictions of the strongest El Niño and La Niña episodes at medium and longer leads. Some of the larger errors seen in Fig. 8 are also for long-lead forecasts made during an ENSO event for conditions to come 4 to 12 months later. For example, during the 1986–1987 El Niño, forecasts for a continuation of El Niño conditions during 1987–1988 did not appear until late summer 1987 when the erroneously predicted return to neutral condition was clearly not occurring. On the other hand, the strong La Niña of 2010–2011 following the El Niño of 2009–2010 was underpredicted at long lead, and its continuation at a weaker level for a second consecutive year (2011–2012) was missed at both intermediate and longer leads. Based on Figs. 7 and 8, it appears that no one particular ENSO situation leads to most of the errors of the MME, and that relatively large forecast errors can be made in a variety of circumstances. Besides errors related to the imperfect physical representations in the models themselves, another error source is in the fields of oceanic initial conditions, using current data assimilation systems (Xue et al. 2017).

It will be shown below that the Northern Hemisphere spring ENSO predictability barrier (e.g., Jin et al. 2008; hereafter called the spring barrier) is a major factor causing generally decreased verification skill for both individual models and the MME. The spring barrier causes forecasts that traverse the months of April through June to tend to have less favorable verification than those that do not go through those months. Because a northern spring season typically separates consecutive ENSO cycles, the spring barrier causes difficulty in predicting the ENSO state 6 to 12 months forward from the peak of an ENSO episode that often occurs near the end of a calendar year.

While Figs. 3, 4, 5, 6, 7 and 8 show details of the predictions, observations and errors one at a time over the 35-year period, we look to summarize the individual model and the MME performances over the entire 35 years in terms of the average size of the errors and the anomaly correlations between predictions and observations.

### Deterministic verification of the NMME predictions

The accuracy of the predictions can be indicated by their MSE with respect to the observations, over the 1982–2015 period. The MSE is defined as the average, for predictions for a given target month and lead time, of the squared differences between the predictions and their corresponding observations. A feature of the MSE that makes inferences of model skill challenging is that the size of the models’ errors tends to parallel the seasonally changing interannual variability of the Niño3.4 SST anomaly. Specifically, the interannual standard deviation of the SST in the Niño3.4 region is between 1.0 and 1.4 °C during late northern autumn and winter months, but only 0.5–0.8 °C in late spring and early summer months. A given MSE would therefore imply a relatively better model performance for predictions for winter than for late spring because one would expect smaller errors given the lower variability in spring. This problem is overcome by translating the MSE into a skill score by comparing it to the MSE expected when making perpetual forecasts for climatology (zero anomaly), as the latter would have larger (smaller) MSE during times of high (low) interannual standard deviation. In fact, the MSE of climatology forecasts equals the standard deviation of the observations. The MSE skill score (MSESS) is defined as

1 $$MSESS=1-~\frac{MS{{E}_{fct}}}{MS{{E}_{c\lim }}}$$

where MSE_fct_ is the MSE of the model forecasts and MSE_clim_ is the MSE of climatology forecasts. When MSE_fct_ is equal in size to MSE_clim_, MSESS is zero.

**Fig. 9:**
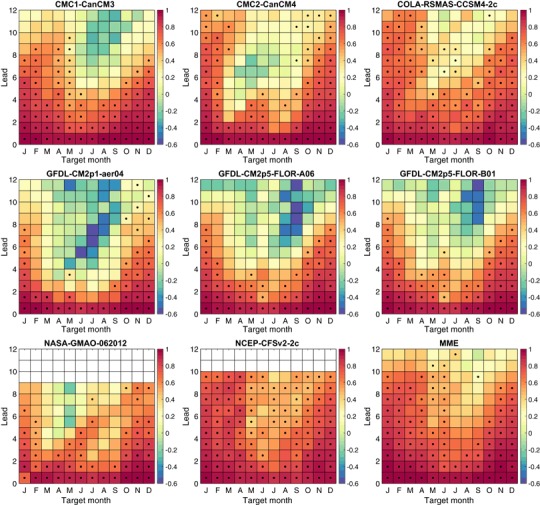
Mean squared error skill score (MSESS) of ensemble mean anomaly forecasts as a function of target month and lead in months. *Black dots* indicate MSESS that is statistically significantly greater than a climatological forecast at 5% significance level, using a sign test

Figure 9 shows the MSESS of each individual NMME model and the MME as a function of target month and lead time. The pattern of MSESS shows, for most lead times for all models and for the MME, relative decreases in skill beginning around April or May and continuing to later months, especially for moderate and long lead forecasts. The explanation for this pattern is that the spring barrier (April through June) is traversed in the lead times of such forecasts, while for shorter lead forecasts there is a recovery in skill for the later calendar months because the forecasts start later than the time of the barrier. The effect of the spring barrier on MSESS is most pronounced in the CMC1 and the three GFDL models, and less severe in the CFSv2, CCSM4, NASA and CMC2 models as well as the MME. A general effect of the barrier at moderate and long lead times is a degradation of MSESS to the point where they are no longer statistically significantly better than forecasts for the climatological average (i.e., zero anomaly). Further, for most of the models MSESS becomes negative at medium to long leads for target months occurring slightly later than the end of the spring barrier. While negative MSESS means that the MSE is larger than the MSE of climatology forecasts, it does not indicate that the forecasts have no information value (e.g., they may have inappropriately high amplitude but phasing in synchrony with observations), as will be shown below using the anomaly correlation as the verification measure.

In addition to showing differences in the impacts of the spring barrier on MSESS among the models, Fig. 9 also shows model differences in MSESS across the target months and leads that are largely not affected by the spring barrier. Examples are the short and intermediate leads for the target months of November through March (far left and right sides of the panels). For these conditions, the CFSv2, CMC1, CCSM4 models and the MME show the most positive and most frequently statistically significant MSESS.

**Fig. 10:**
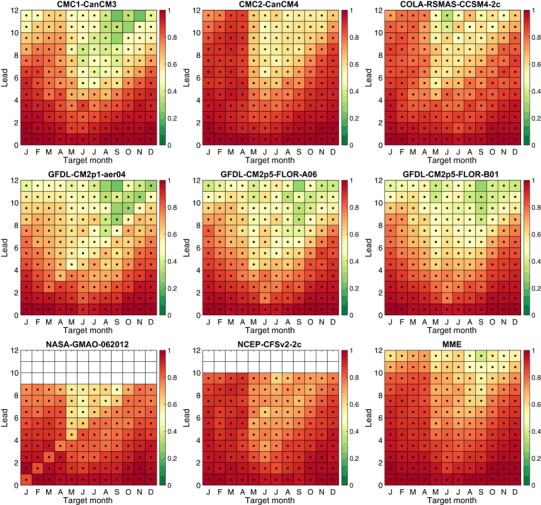
Anomaly correlation of ensemble mean forecast and observed anomalies as a function of target month and lead time in months. *Black dots* indicate correlation values that are statistically significantly greater than zero at 5% significance level, using a *t* test

Figure 10 shows the temporal anomaly correlation between model forecasts and observations as a function of target month and lead. The correlation can be considered a measure of basic predictive potential, in that it shows discrimination ability and is not affected by the calibration issues (e.g., mean bias and amplitude bias) that degrade MSESS. There is a tendency for a general correspondence between models showing overall high MSESS (Fig. 9) and high correlation (Fig. 10), as in the case of CFSv2, and low MSESS and correlation, such as seen in GFDL. The MME tends to perform at the correlation level of the one or two models with highest correlations. While the patterns of correlation roughly resemble those of MSESS, the magnitudes and the instances of statistical significance are greater for the correlation because biases in amplitude decrease the MSESS, while they do not affect the correlation.^3^ Thus, the correlations are always positive and nearly always statistically significant, while the MSESS is significant for only about half of the target/lead combinations for some models, and in some cases it is negative. The pervasive presence of significant correlations (here, correlations of at least 0.35) implies that the models usually have useful discrimination in predicting the interannual variability of tropical Pacific, even at the longest lead times for most models, but they have amplitude biases that prevent their MSESS from being comparably statistically significant. If desired, such amplitude biases could be removed using a linear statistical adjustment, as attempted in Barnston et al. (2015). Such a linear rescaling has been applied to the real-time NMME predictions of Niño3.4 anomaly shown in NOAA/Climate Prediction Center’s page: http://www.cpc.ncep.noaa.gov/products/NMME/current/plume.html.

The degree to which calibration errors reduce MSESS, resulting in lower skill than that corresponding to the correlation in their absence, can be determined through a decomposition of the MSESS as detailed in Murphy (1988). The relevant equation, showing the three components of MSESS is:

2 $$MSESS=co{{r}^{2}}-{{\left( cor-\frac{S{{D}_{fct}}}{S{{D}_{obs}}} \right)}^{2}}-{{\left( \frac{mea{{n}_{fct}}-mea{{n}_{obs}}}{S{{D}_{obs}}} \right)}^{2}}$$

Equation (2) shows that the MSESS is governed by three components: the anomaly correlation (as shown in Fig. 10), the amplitude bias, and the mean bias. The square of the correlation establishes the upper limit of MSESS, as the amplitude bias and mean bias can only degrade MSESS. An amplitude bias exists when the ratio of the standard deviation of the forecasts to that of the observation deviates from the correlation. For example, if there is no amplitude bias, and the correlation is 0.5, then the standard deviation of the forecasts should be half that of the observations. Such damping of the forecasts minimizes their MSE. If it is more than half, the forecasts are “overconfident” relative to their underlying correlation skill, and if less than half they are “underconfident”.

**Fig. 11:**
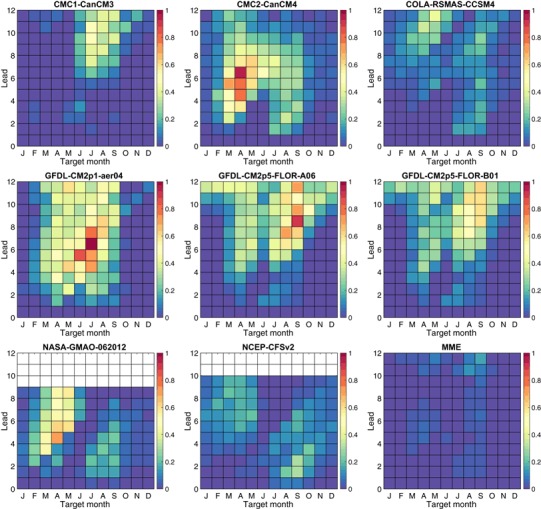
Squared amplitude bias as a component of the decomposition of the MSESS. Here the CFSv2 and CCSM4 models are analyzed with a single climatology instead of the dual climatologies as in the other analyses. See text for details

To isolate the contribution of amplitude bias to the MSESS, Fig. 11 shows the squared amplitude bias (second term on the right side of Eq. (2)) by target month and lead, for each model and for the MME. Here, the CFSv2 and CCSM4 models are analyzed with a single climatology instead of the dual climatologies as in the other analyses.^4^ Examination of the sign of the amplitude bias (not shown) indicates that in virtually all cases the bias is due to too-high forecast amplitudes rather than too-low amplitudes. The largest amplitude biases are seen mainly for medium and long lead forecasts for months in the middle of the calendar year, as noted particularly in the CMC2, GFDL and GFDL-FLOR-A models. These target/lead combinations are quite congruent with those having lowest MSESS in the same models (Fig. 9), despite that CMC2 largely escapes a low MSESS because of its relatively high correlation skill (Fig. 10). Some studies have indicated that amplitude biases in individual model ensemble mean predictions are usually due to forecast amplitudes larger than warranted by the correlation, particularly for target seasons and leads that have relatively poor predictive skill due to the spring barrier (e.g., Barnston et al. 2015). The amplitude biases here tend to confirm this, although for some models the instances of highest amplitude bias shown in Fig. 11 occur slightly earlier in the calendar year than would be expected for forecasts affected solely by the spring barrier. A possible additional cause of the amplitude bias could be a failure of some models to reproduce the decreased observed interannual variability of the SST in late spring. The months of minimum variability are April to June, which is close to the month of largest amplitude bias in CMC2, GFDL and NASA. It is then possible that the amplitude bias is greatest mainly at the beginning of the extended period during which forecasts traverse the spring barrier (for medium and longer lead)—namely, that portion when the observed interannual variability is near its minimum.

The MME is remarkably free of amplitude bias (Fig. 11), given that the individual models having the least amount of this bias—CFSv2 and CCSM4 (even without the dual climatology adjustment)—nonetheless have relatively more of it. This is presumably due to the cancellation effect of differing predicted anomalies from models that may individually be overconfident. The beneficial calibrating effect of forming the MME, also noted in Barnston et al. (2015), is a strong selling point for it.

**Fig. 12:**
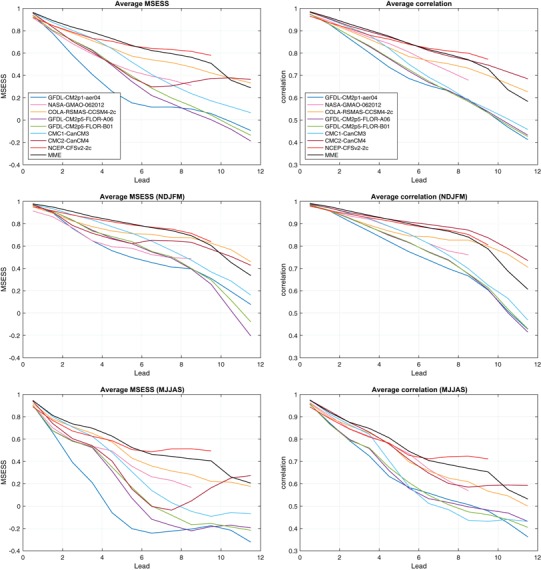
Mean-squared error skill score (MSESS; *left*) and anomaly correlation (*right*), as a function of lead and averaged over (*top* row) all 12 target months, (*middle* row) the better forecast target months of November through March, and (*bottom* row) the more challenging forecast target months of May through September. The Fisher-Z transform is used in averaging the correlations

To further summarize the relative model performances, graphs of seasonally averaged MSESS and anomaly correlation are shown in Fig. 12. The left side of Fig. 12 shows MSESS for the individual models and the MME, as a function of lead time. MSESS is shown averaged over all months (top panel), for just November through March (the more predictable time of year; middle panel), and for just May through September (the less predictable time of year; bottom panel). The differences in MSESS among models are substantial, especially for middle and long leads. For all months together, the CFSv2, CCSM4 and MME show highest values for many of the leads, while the three GFDL models show lowest MSESS. The CMC1 model is also in the group of best performers at short leads but drops to a more middle rank at longer leads, while the CMC2 is at a middle rank at short leads but moves to the top-ranked group at the longest leads. The MME shows the best MSESS for short leads, but is surpassed by CFSv2 from 6.5-month lead until its final lead at 9.5 months. The MME drops to third place for 10.5- and 11.5-month leads, likely influenced by the three GFDL models whose MSESS becomes negative as well as by the discontinued support from the CFSv2 model. The seasonally stratified results clearly show the skill-impeding effect of the spring barrier for forecasts for May through September, compared with the more favorable skills for November through March. The seasonally stratified results show that the rising rank of CMC2 occurs most clearly in its May–September forecasts, in which there is a remarkable actual improvement in MSESS from 7.5-month lead through the final 11.5-month lead. This “return of skill” in CMC2 may or may not have physical causation.

Seasonally averaged temporal anomaly correlation results are shown on the right side of Fig. 12, again averaged over all target months (top panel), for November through March (middle panel), and May through September (bottom panel). The Fisher-Z transform is used in averaging the correlations. For all months together, the CFSv2, CMC2 and MME show highest correlation for most leads, with CCSM4 and NASA just below them. The three GFDL models and the CMC1 show lowest correlation. The seasonally stratified results show similar ranking patterns. For all months combined, the MME and the CMC2 have the best correlations through 5.5-month lead. For 6.5 and 7.5-month leads, the MME is outperformed by CFSv2, and at 8.5-month lead by both CFSv2 and CMC2, and at 10.5 and 11.5-month leads it is lower than CCSM4 as well. The relative drop in the MME at longest lead times is partly due to the discontinuation of CFSv2 after 9.5-month lead.

The all-months and the seasonally stratified correlation graphs in Fig. 12 show roughly similar shapes to their MSESS counterparts for the November through March result, but noticeable differences appears in the May through September graph, and also to some degree in the all-months graph. For the more challenging target months of May through September, for many of the models there is a drop in MSESS for the middle lead times (e.g., 5.5–8.5 months) that does not appear in the correlation results. This difference is related to the tendency for some models to make predictions of higher amplitude than is appropriate during relatively low skill conditions, such as predictions for the third quarter of the year made before May (i.e., traversing the spring barrier). As discussed above in the context of Eq. (2), the MSESS is maximized when the predictions follow the “regression rule” of squared error minimization, calling for predictions to be damped (have smaller amplitude) in proportion to the absence of correlation skill for the given target month and lead time. The negative MSESS seen in the predictions for May to September at middle or long lead times for the three GFDL models and the CMC1 model (Fig. 12, lower left panel), but positive associated correlations (Fig. 12, lower right panel) are caused by “overconfident” forecast amplitudes (Fig. 11) that increase squared errors.

The imperfect calibration implied for some of the individual models during the more challenging target months is greatly reduced in the MME predictions (Fig. 12). This observation is consistent with the favorable finding of good probabilistic reliability of the MME in Tippett et al. (2017; see their Fig. 12). As mentioned earlier, a well-calibrated MME is possible even with inflated amplitudes in some of the individual models, because the effects of the predictions of those models are often reduced due to cancellation in the MME.

The all-season correlation skills for the MME and its two strongest individual model competitors (CFSv2 and CMC2) shown in Fig. 12 are shown more precisely in Table 2, along with the average correlation skill^5^ across all individual models (labeled AVG:NMME). The MME has highest skill for 0.5- to 4.5-month leads, CMC2 is highest at 5.5-month lead, followed by CFSv2 out to its longest lead of 9.5 months. This outcome is consistent with the finding in Kirtman et al. (2014) for SST and climate variables—namely, that some individual models may be superior to the MME in certain locations, seasons and lead times, but the MME is always close to being top-ranked when it is not so, and generally has skill well above that of the average of the individual model skills. The bottom line in Table 2 confirms that the average of the individual model anomaly correlations is below the MME result at all leads.

**Table 2:** Anomaly correlation performance, over 1982–2015, of the MME and the most skillful two individual models: CMC2 and CFSv2

1982–2015	Lead (months)											
	0.5	1.5	2.5	3.5	4.5	5.5	6.5	7.5	8.5	9.5	10.5	11.5
MME	**0.985**	**0.959**	**0.929**	**0.903**	**0.875**	0.843	0.815	0.79	0.77	0.73	0.64	0.58
CMC2	0.983	0.953	0.921	0.896	0.871	**0.844**	0.811	0.79	0.77	0.75	**0.72**	**0.68**
CFSv2	0.966	0.935	0.910	0.880	0.862	0.839	**0.821**	**0.81**	**0.80**	**0.77**		
Avg:NMME	0.975	0.938	0.90	0.86	0.82	0.77	0.73	0.70	0.67	0.63	0.56	0.52

The bottom row shows the average of the anomaly correlation across the individual models of the NMME—i.e., the average of the model skills, not the skill of the average of the forecasts as in the MME. Hindcasts (but real-time forecasts for 2011–2015) are for 1-month average SST. For each lead time, the highest skill result is shown in bold. The Fisher-Z transform is used in averaging the correlations in bottom row

### How skillful is the NMME compared with other recent model sets?

In Sect. 1, it was stated that the NMME model set contains some, but not all, of today’s best models. How can we show that this model set is among today’s best? One avenue for such a demonstration is a comparison of NMME skills to those of other model sets examined in recent or older ENSO prediction skill analyses. In Barnston et al. (2012), anomaly correlation skills were computed for real-time forecasts of 3-month average Niño3.4 SST shown on the “IRI/CPC ENSO prediction plume” (hereafter called “the plume”) for the 2002–2011 period, and also for longer-term hindcasts from some of the same models. When available, longer-term hindcast skills from the plume covered the period 1981–2010. The first column in Table 3 identifies the seven plume models that provided hindcasts. Five out of the seven models are comprehensive coupled models, comparable to the NMME models, while two of them (LDEO and KMA-SNU) are not so (Table 3). To compare the skills of the seven hindcasts on the plume with those of the NMME, we compare the respective averages of the skills of their individual models. For this comparison, NMME model hindcasts of 3-month average SST were used, and the period of 1982–2000 was used to best match the plume hindcast period. Table 4 shows skill results for the comparison, averaged over all seasons and models, where the average is shown both for the skills of the seven dynamical models on the plume (in the row labeled “Plume-7”) and for only the five comprehensive dynamical models (labeled “Plume-5”). While the average skills that omit the non-comprehensive models are slightly higher than those in which they are included, the average of the NMME models’ skills (top row of Table 4) is slightly higher than that of the plume’s comprehensive model skill at all leads. This suggests that the NMME skills are likely superior to, or at least competitive with, those of comparable models of the recent past. A caveat pertinent to this skill comparison relates to the small sample size of models used for the plume hindcasts (only five comprehensive coupled models), likely leading to sampling variability in the estimates of average skill.

**Table 3:** Models on the IRI/CPC ENSO prediction plume included in each of the rows in Tables 4 and 5

Dynamical model name on IRI/CPC ENSO prediction plume	Model type	Table 4 Hind Avg: Plume-7	Table 4 Hind Avg: Plume-5	Table 5 RT MME Plume-15	Table 5 RT Avg: Plume-12	Table 5 RT Avg: Plume-8
		Barn12	Barn12	Tipp12	Barn12	Barn12
NASA Global Modeling and Assimilation Office (GMAO)	Fully coupled			X	X	X
NCEP Climate Forecast System (CFSv2)	Fully coupled	X	X	X	X	X
Japan Meteorological Agency (JMA)	Fully coupled			X	X	X
Scripps Hybrid Coupled Model	Comprehensive ocean, statistical atmosphere			X	X	
Lamont-Doherty Earth Observatory (LDEO)	Intermediate coupled	X		X	X	
Predictive Ocean Atmosphere Model for Australia (POAMA)	Fully coupled	X	X	X	X	X
European Center for Medium-Range Weather Forecasts Model (ECMWF)	Fully coupled			X	X	X
United Kingdom Meteorological Office (UKMO)	Fully coupled			X	X	X
Korea Meteorological Administration, Seoul National University (KMA-SNU)	Intermediate coupled	X		X	X	
Univ. of Maryland Earth System Science Interdisci-plinary Center (ESSIC)	Intermediate coupled			X	X	
IRI ECHAM/Modular Ocean Model: Anomaly	Anomaly coupled	X	X			
IRI ECHAM/Modular Ocean Model: Direct	Fully coupled	X	X			
IRI ECHAM/Modular Ocean Model: Direct and Anomaly	Anomaly and fully coupled			X	X	X
Center for Ocean-Land-Atmosphere Studies (COLA)	Anomaly coupled			X	X	X
COLA Community Climate System Model, version 3 (CCSM3)	Fully coupled			X		
Meteo-France	Fully coupled	X	X	X		
Japan Frontier Research Center for Global Change	Fully coupled			X		

In column headings, *RT* real-time, *Hind* hindcasts

The second row indicates the publication source: Tipp12 is for Tippett et al. (2012), Barn12 is for Barnston et al. (2012)

**Table 4:** Top 3 rows: Anomaly correlation skill, averaged* across models and seasons, for the hindcasts of the NMME (8 models), the seven available dynamical models in the IRI/CPC ENSO prediction plume (Plume-7), and the same but without the two intermediate or 2-tiered dynamical models (Plume-5)

1981(2)–2010	Lead (months to center of 3-mon target season)									
	1.5	2.5	3.5	4.5	5.5	6.5	7.5	8.5	9.5	10.5
Avg: NMME hind	**0.948**	**0.91**	**0.87**	**0.82**	**0.78**	**0.75**	**0.72**	**0.67**	**0.63**	0.58
Avg: Plume-7 hind	0.928	0.88	0.83	0.79	0.74	0.71	**0**.68	0.61	0.56	
Avg: Plume-5 hind	0.935	0.89	0.84	0.80	0.75	0.73	0.69			
MME: NMME hind	0.967	0.94	0.91	0.88	0.86	0.83	0.81	0.78	0.70	0.65

The NMME data spans 1982–2010 while the plume data is for 1981–2010. Both sets of hindcasts are both for 3-month mean Niño3.4 SST anomaly. For each lead time, the highest skill result among the first three rows is shown in bold. See Table 3 for the sets of models included in the first three rows. The bottom row shows the skill of the ensemble mean forecasts from the NMME, in contrast with the top row that shows the average of the skills of the individual NMME models

*The Fisher-Z transform is used in averaging the correlations

**Table 5:** Skill results for 3-month averages for the subperiod 2002–2011

2002–2011	Lead (months to center of 3-mon target season)									
	1.5	2.5	3.5	4.5	5.5	6.5	7.5	8.5	9.5	10.5
MME: NMME hind	**0.974**	**0.95**	**0.92**	**0.89**	**0.85**	**0.80**	**0.76**	**0.71**	**0.64**	0.57
MME: Plume-15 RT	0.962	0.93	0.90	0.85	0.81	0.75	0.69	0.63	0.46	
Avg: NMME hind	**0.960**	**0.93**	**0.90**	**0.86**	**0.82**	**0.78**	**0.73**	**0.66**	0.61	0.55
Avg: Plume-12 RT	0.917	0.87	0.82	0.76	0.66	0.54	0.45	0.39		
Avg: Plume-8 RT	0.929	0.89	0.85	0.80	0.69	0.52	0.41	0.35		

*Top two rows* Anomaly correlation skill of the average of the hindcasts of the 8 NMME models (i.e., the MME), averaged across seasons*, and likewise for the real-time predictions of the IRI/CPC ENSO forecast plume’s dynamical models (15 models; from Tippett et al. 2012). *Bottom 3 rows* Anomaly correlation skill, averaged* across models and seasons, for the NMME (8 models), the IRI/CPC ENSO forecast plume’s 12 dynamical models having forecast data for at least 60% of the start times (Plume-12; from Barnston et al. 2012), and the same but without the four intermediate, 2-tiered or hybrid dynamical models (Plume-8). For each lead time, the highest skill result is shown in bold within the top two rows and within the bottom three rows. See Table 3 for the sets of models included in the results in each row

*The Fisher-Z transform is used in averaging the correlations

The bottom line in Table 4 shows the skill of the MME for predictions of 3-month average SST during 1982–2010, to be compared with the top line that averages the individual model skills. As expected, the MME skill is higher than the average of the model skills. The 0.94 skill for 2.5-month lead MME predictions is similar to the skill for that lead (0.93) found in Becker et al. (2014) despite that their NMME model set was slightly different (e.g., it included NCEP CFSv1 and COLA-RSMAS CCSM3).

Another way to determine whether the NMME model set contains some of today’s best ENSO-related SST prediction models, is to compare its skills to those of real-time predictions of Nino3.4 SST anomalies shown in the same plume mentioned above, over the 2002–2011 period as examined in two recent studies: Real-time MME predictions from the plume models are examined in Tippett et al. (2012), and comparisons among individual plume models are provided in Barnston et al. (2012). The two studies therefore allow for skill comparisons with the NMME both in terms of the skill of the averaged forecasts (i.e., the MME) and the averages of the skills of the individual models. Because there are marked decadal fluctuations in the difficulty of making ENSO predictions (Barnston et al. 2012), a fair comparison requires that the skill of the NMME hindcasts be examined for the same 2002–2011 period as covered by the prediction plume studies. That the predictions in the prediction plume are made in real-time while the NMME predictions are hindcasts (with the exception of those made in 2011) represents an inconsistency in the comparison that likely gives a slight advantage to the NMME.

Table 5 shows comparative skills for the reduced 2002–2011 period. The top two rows compare the skills of the MME hindcasts of 3-month average SST from the NMME models, and the MME from the real-time forecasts of 3-month average SST of 15 models on the IRI/CPC ENSO prediction plume. Because the forecasts are for 3-month periods, the lead time is keyed to the middle month so that 1.5 months is the shortest lead. Accepting the hindcast versus real-time forecast difference, we find that the NMME has slightly higher correlation skills for the shortest leads, with this difference increasing with increasing lead times. Looking at the list of models in the plume MME (Table 3, third column from right), four of the 15 models are seen to be either intermediate coupled (LDEO, KMA-SNU, and ESSIC) or hybrid coupled (Scripps). These models with non-comprehensive oceanic and atmospheric physics might be expected to have lower skills than the models labeled “fully coupled”.^6^

It is desirable to compare MME skills between the NMME and the plume models excluding the non-fully coupled models. But an MME from this dynamical model subset is not evaluated in Tippett et al. (2012). As an alternative comparison, the average of the correlation skills of the individual NMME models and two choices of the subset of ENSO prediction plume models are shown in the bottom three rows of Table 5.^7^ As expected, the average of the skills of the NMME models (top row of the bottom three rows) is lower than the skill of the average of the NMME model forecasts (top row of Table 5)—a selling point for the use of MME. In the comparison between the average of the NMME model skills with the average of the skills of the 12 dynamical models whose real-time forecasts were evaluated in Barnston et al. (2012), the NMME models deliver substantially higher correlations for all leads. With the four non-fully coupled models removed from the plume’s skill average (“Avg:Plume-8” in Table 5), the NMME models still show an advantage, but somewhat less strongly. Again, the slight advantage of the mainly hindcasts of the NMME over the real-time predictions of the plume models should be kept in mind.

One can select a smaller group of the highest performing models on the prediction plume in an attempt to outperform the average of the 8 NMME model skills. For example, using only the four best plume models (ECMWF, CFSv2, Japanese Meterological Agency and ECHAM/MOM models), brings the average skills close to those of the NMME models for most leads (not shown). But if only the four highest performing NMME models were used, a similar increase in the skill average would be expected, and the comparison becomes increasingly aimed at finding the best single model in each group rather than the best set of models, or the set of models that would yield the best MME skill rather than the best average skill. Also, in practical situations, identifying the best model(s) may not be obvious a priori without doing analyses such as those done here. The MME skills of the NMME models versus the MME of another group of approximately the same number of models is the comparison desired, and that shown in the top two rows of Table 4 is our best approximation here, as it compares two MMEs. This result, along with the fact that the 8-model NMME has skill average (third to bottom row of Table 5) considerably greater than that of the 8-model prediction plume (bottom row of Table 5) place the NMME models in a favorable light.

The above comparisons raise the question of whether the skill of an MME, or alternatively the average of the skills of a set of models, depends simply on the skills of the constituent models. Within the NMME model set, for example, the CFSv2 and CMC2 models have been shown to be the skill leaders in many of the analyses using the 1982–2015 hindcasts and real-time predictions; similarly, in the plume, four of the fully coupled models models were shown to have highest correlation skill during 2002–2011. Perhaps determining which MME of those in current existence [e.g. the NMME, a Eurosip MME, a World Meteorological Organization (WMO) MME, or others] is the most skillful is depends mainly on the skills of each group’s individual models. On the other hand, inter-model complementarity may also be important. Thus, in Kirtman et al. (2014) it was argued that the skill of a MME is determined not only through the average skills of its constituent models, but also by the complementary nature of the models’ contributions. Assessment of the degree of complementarity in a set of models—resulting in a skill improvement beyond that expected due to the larger ensemble size alone—is a subject of interest in its own right, but is beyond the scope of this study.

## Discussion and conclusion

The quality of the NMME predictions of ENSO-related east-central tropical Pacific Ocean SST anomaly is examined, and performance findings are compared with those of other recent ENSO prediction skill studies. Verifications are done on individual models in the NMME as well as on the MME forecasts. Here, only the deterministic predictive skill of the NMME is addressed, as a probabilistic evaluation of the NMME predictions is presented in Tippett et al. (2017). The NMME predictions consist of hindcasts during the period 1982 to 2010, and real-time forecasts from 2011 to 2015, covering target months into 2016 during the dissipation of the strong 2015–2016 El Niño.

The two verification measures used here are the mean squared error skill score (MSESS) and the temporal anomaly correlation. Verification of individual models shows somewhat differing skills among the 8 NMME models included, with some models consistently producing more successful predictions than others. Across varying times of the year and lead times, the top two performing individual models are found to be the NOAA/NCEP CFSv2 and the Canadian CMC2 models. The MME predictions are at approximately the same skill levels as these two best performing individual models.

Decomposition of the MSESS (as detailed in Murphy 1988) suggests that calibration errors are present in most of the models, but most notably in certain ones. Revealed is a tendency toward overly confident (i.e., too-high amplitude) forecasts in predictions made prior to the northern spring predictability barrier for targets near or shortly following the barrier. Overly strong predictions made in conditions of low expected skill fail to minimize squared errors, so that the MSESS scores are decreased below their optimum level considering the anomaly correlation. For example, for some target months and leads, models with statistically significant correlation skill (e.g., 0.5) have negative MSESS, indicating that even perpetual predictions of the climatological average would have smaller squared errors, and predictions of smaller amplitude would have positive MSESS. In addition to too-high amplitude in predictions traversing the spring barrier, forecast amplitude may be too high for predictions for target months whose interannual variability is near the seasonal minimum—April, May and June—as some models do not effectively reproduce the seasonal cycle of the interannual variability. (Note that the times of the spring barrier and the minimum in interannual variability are roughly the same.)

The skill of the NMME system is compared to the skill of the MME from the IRI/CPC ENSO prediction plume, both for a comparable 3-decade long hindcast period and for a set of real-time predictions during 2002–2011. Comparisons are made not only between MME predictions of the NMME and the plume, but also between the average of the skills of the individual models in each group. In these comparisons, in which 3-month average SST anomalies are used, the skill of the NMME’s MME is found to be slightly higher than that of the prediction plume both for the long-period hindcasts and for the 9-years of recent real-time plume predictions. However, the recent real-time plume predictions may be more challenging than the comparable NMME predictions, which are mostly hindcasts. Accepting this imbalance, the results still reflect well on the MME predictions. A discussion of the top performing models in each of the NMME set and the plume set emphasizes that each set has its better and its worse performers, but that a feature of MME predictions is complementarity—that they benefit from the strengths of each of the models, that may alternate in differing circumstances, and that the differing forecasts of the poorer models in any situation tend to cancel.

## Footnotes

1 The ECMWF model was found to have the highest forecast skill for real-time forecasts during 2002–2011 among the 15 dynamical models included in Barnston et al. (2012).

2 Removal of mean bias is not fully accomplished because the study period includes 1982–2015, as 5 years of real-time forecasts are included. .

3 Mean bias also would also decrease MSESS (and not correlation), but because the predictions are anomalies with respect to the models’ own means—although only for the majority hindcast portion of the period—mean biases are largely eliminated by design. Accordingly, mean biases are found to be very small (less than 0.1 °C for most target/lead combinations; not shown).

4 Single climatologies are used here to place all models on an equal footing, given that using two differing climatologies decreases the amplitude bias.

5 In this study, correlations are averaged using the Fisher r-to-Z transformation, where the Fisher Zs are averaged and the result is transformed back to correlation (r).

6 In Barnston et al. (2012) the intermediate coupled models, as a group, showed lower performance than the group of fully coupled models.

7 In Tables 2, 4 and 5, the distinction between the skill of the forecast average and the average of the skills of individual models’ forecasts (using the Fisher Z to average the correlations) is clarified by the row headings, using “MME” for the former, and “AVG:” for the latter.
